# A Rare Cause of Haemorrhagic Shock: Rupture of Gastric Wall Seeding of Hepatocellular Carcinoma

**DOI:** 10.1155/2022/6560834

**Published:** 2022-03-12

**Authors:** Naoki Ishimaru, Hirohisa Fujikawa, Kazuya Niwa, Yoshifumi Kobayashi

**Affiliations:** ^1^Department of Surgery, Suwa Central Hospital, 4300 Tamagawa, Chino, Nagano 3918503, Japan; ^2^Department of Medical Education Studies, International Research Center for Medical Education, Graduate School of Medicine, The University of Tokyo, 7-3-1 Hongo, Bunkyo-ku, Tokyo 1130033, Japan; ^3^Department of Internal Medicine, Suwa Central Hospital, 4300 Tamagawa, Chino, Nagano 3918503, Japan

## Abstract

Ruptured hepatocellular carcinoma (HCC) can lead to peritoneal dissemination. However, gastric wall seeding from HCC is exceedingly rare, and little is known about its clinical course. Herein, we report a case of an 88-year-old man who presented with a four-hour history of nausea, vomiting, and upper abdominal pain. He has a history of ruptured HCC during surgery. The patient underwent an emergency laparotomy on account of haemorrhagic shock, which confirmed the diagnosis of ruptured HCC with gastric wall seeding. The findings from this study showed that the ruptured HCC can seed into the stomach wall, and the implanted lesions may rupture and lead to life-threatening haemorrhagic shock. Surgery is an effective treatment for bleeding from the implanted lesions.

## 1. Introduction

Although great progress has been made in the treatment of hepatocellular carcinoma (HCC) in recent years, recurrence rate is still very high even after curative therapy [1]. Extrahepatic recurrence of HCC is usually caused by haematogenous metastasis [2]. Peritoneal seeding of HCC into the gastric serosa after HCC rupture is rare [3]. Moreover, treatment strategies for cases where the seeded lesions rupture have not been well established. Herein, we describe a rare case of ruptured gastric wall seeded HCC, leading to severe haemorrhagic shock. We opted for surgery, which helped save the patient's life.

## 2. Case Presentation

An 88-year-old man with chronic kidney disease presented to our hospital with a four-hour history of nausea, vomiting, and upper abdominal pain. He exhibited no symptoms of hematemesis, melena, or anaemia. Fifteen months prior to presentation, he underwent surgical dissection with lateral hepatectomy due to HCC. The liver tumour ruptured during the surgical procedure. We washed the peritoneal cavity with 5 litres of normal saline to remove the tumour cells and reduce the risk of dissemination. Nine months earlier, abdominal computed tomography (CT) had revealed a nodule (10 mm) on the gastric antrum's serous surface. Three months later, the nodule size was not remarkably different, as confirmed by abdominal ultrasonography and CT. Due to the patient's advanced age and history of renal dysfunction, adjuvant chemotherapy was not administered.

**Figure 1:**
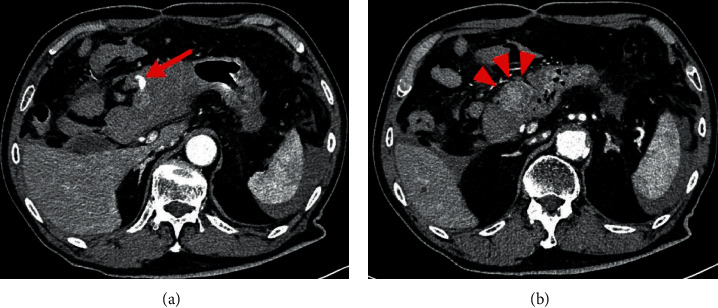
Contrast-enhanced abdominal computed tomography showed contrast leakage ((a), arrow) into the peritoneal cavity from a hypervascular mass ((b), arrowheads, 30 mm) on the gastric antrum's anterior wall.

He was pale on examination with blood pressure (85/55 mm Hg), pulse rate (102 bpm), and temperature (34.9°C). He had notable tenderness in the epigastrium. Laboratory investigations showed elevated white cell count (10.1 × 10^9^/L), while other findings were unremarkable. Contrast-enhanced abdominal CT showed contrast leakage into the peritoneal cavity from a 30 mm mass on the gastric antrum's anterior wall (Figure 1). Haemorrhage was suspected, and an emergency laparotomy was performed, which revealed about 800 mL of blood in the peritoneal cavity. A gastric antrum tumour with persistent bleeding protruded from the anterior wall. The gastric serosa and tumour were resected using a vascular sealing device. We then performed wedge resection of the tumour. Since the muscularis propria of the stomach was preserved and no bleeding was observed, the gastric serosa's membrane defect was sutured.

**Figure 2:**
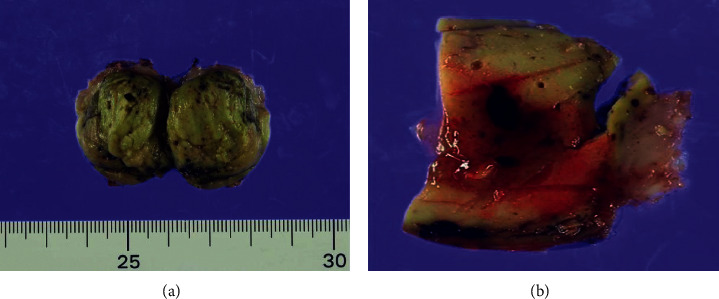
Macroscopic findings. (a) Greenish and soft tumour that measured 30 × 30 × 25 mm in size. (b) The tumour was haemorrhagic solid.

**Figure 3:**
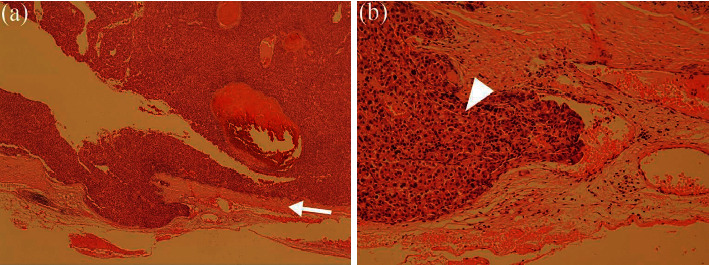
Microscopic pathological examination of the tumour. (a) There was no obvious gastric serosa (arrow) exposure of the tumour (haematoxylin and eosin stain, original magnification ×20). (b) Moderately differentiated hepatocellular carcinoma (arrowhead) (haematoxylin and eosin stain, original magnification ×100).

Macroscopically, the tumour was greenish, haemorrhagic, solid, and soft and measured 30 × 30 × 25 mm in size (Figure 2). Histopathological examination of the tumour revealed a moderately differentiated HCC (Figure 3). There was no indication of gastric serosa exposure to the tumour, and the surgical margins were negative. Based on these findings, a diagnosis of HCC seeding was made. The patient was discharged 18 days after surgery without any complications. According to the previous reports, postoperative chemotherapy and the use of molecular targeted drugs for hepatectomy are ineffective in preventing recurrence [4, 5]. After discussing with the patient, it was decided that adjuvant chemotherapy would not be initiated considering the published evidence, the patient's age, and poor performance status. CT surveillance is performed every 3 months since surgery; no recurrence has been observed till date (6 months since surgery).

## 3. Discussion

HCC can lead to peritoneal dissemination (5.6%) [6]. In this patient, a ruptured HCC had previously been implanted into the gastric wall, and this time, the implanted lesion ruptured, resulting in haemorrhagic shock. Emergency laparotomy was performed to save the patient's life.

The most common type of primary liver cancer is HCC. It usually develops in the setting of underlying chronic liver disease, especially in patients with cirrhosis due to chronic hepatitis B and/or C virus infection, excessive alcohol intake, and nonalcoholic fatty liver disease [7]. Despite treatment advancements, recurrence rate of HCC is still very high. The most frequent recurrence site is the liver. Extrahepatic metastases most commonly involve the lungs, lymph nodes, bones, and adrenal glands [2, 3, 6].

In many cases of postoperative recurrence after resection of HCC, tumour recurrence is seen in the remaining liver tissue [6]. While extrahepatic recurrence of HCC is usually caused by haematogenous metastasis, lymphatic and disseminated metastasis is rare [2]. An important HCC feature is its ability to rupture, leading to potentially life-threatening complications: haemorrhagic shock, liver failure, and peritoneal dissemination. The median survival time after HCC rupture is approximately 4-5 months [8], but dissemination of HCC is rarely a clinical problem.

The present case shows that gastric metastatic implantation can occur and the lesions can rupture. Gastric metastasis from HCC is rare [9, 10], and most of the reported cases are either caused by haematogenous metastasis or direct invasion [11]. However, in a previous autopsy case series, there were 4/181 (2.2%) cases of peritoneal seeding of HCC in the gastric serosa [3], which indicated the possibility that HCC might seed asymptomatically into the gastric serosa. This is the first case in which gastric serosa seeding by HCC was found after the HCC ruptured in the survivor. The present case is significant, and it demonstrated that seeded lesion rupture in the gastric wall can lead to fatal haemorrhagic shock. On the other hand, given the course of the prior surgery, we probably should have suspected that the 10 mm nodule that appeared on the follow-up CT might be a disseminated lesion. We should have performed an 18F-fluorodeoxyglucose positron emission tomography examination and considered early treatment.

For the treatment of extrahepatic metastases of HCC, effective molecular targeted drugs and surgery have been reported when the patient has a good Eastern Cooperative Oncology Group Performance Status and a class A Child–Pugh score [7]. The treatment for bleeding from HCC seeding has not been determined; however, the therapeutic options are transcatheter arterial embolization (TAE) and surgical haemostasis [8]. The TAE is a minimally invasive and standard treatment for intra-abdominal haemorrhage without shock. However, where TAE cannot control bleeding, the patient may risk delayed emergency haemostasis, possibly leading to worsening of haemorrhagic shock [12]. The TAE can also cause embolization site ischaemic necrosis. In this case, since contrast-enhanced CT could not identify the vessels feeding the tumour, it was difficult to perform TAE; hence, laparotomy was performed.

## 4. Conclusion

In conclusion, we have reported a case of rupture of an HCC lesion seeded in the gastric wall causing haemorrhagic shock in a patient with a history of tumour rupture during HCC surgery. Emergency laparotomy saved the patient's life. HCC can cause peritoneal dissemination but rarely causes dissemination into the gastric wall. Clinicians should consider the possibility of rupture of implanted lesions. Surgery is an effective therapeutic option in cases with haemorrhagic shock due to rupture of the implanted lesions.
